# Prenatal diagnosis of lingual cyst and spontaneous regression before birth: A rare case report and literature review

**DOI:** 10.1097/MD.0000000000017873

**Published:** 2019-11-15

**Authors:** Yixiu Zhang, Yunshu Ouyang, Hua Meng, Xining Wu, Zihan Niu

**Affiliations:** Department of Ultrasound, Peking Union Medical College Hospital, Chinese Academy of Medical Sciences and Peking Union Medical College, Beijing, China.

**Keywords:** lingual cyst, prenatal diagnosis, ultrasound

## Abstract

**Rationale:**

We report a case of fetal lingual cyst that was diagnosed prenatally using 2-dimensional and 3-dimensional images during routine second trimester screening. To the best of our knowledge, this is the first description of a cystic lesion disappearing before birth.

**Patient concerns:**

A 36-year-old woman at 22 weeks’ gestation showed the presence of an oral cystic lesion in routine second trimester ultrasound screening. The lesion measured 18 × 15 × 15 mm.

**Interventions:**

A follow-up ultrasound examination was performed every 4 to 6 weeks.

**Outcomes:**

The cyst disappeared in a follow-up ultrasound examination at 35 and 37 weeks of gestation. A male newborn who weighed 3480 g was delivered with no feeding difficulties. The boy was followed to 6 years after birth. The child had normal growth and development, and there was no recurrence of the cyst.

**Lessons:**

Prenatally diagnosed lingual cysts are uncommon findings that can include many different pathologies with a wide spectrum of severity. Lingual cysts usually have a good prognosis.

## 1 Introduction

Lingual cysts are uncommon findings that can include many different pathologies with a wide spectrum of severity. Prenatal diagnosis of a lingual cyst has rarely been reported.^[1]^ We describe a case of fetal lingual cyst that was diagnosed prenatally. Additionally, a follow-up examination showed that the cystic lesion disappeared before birth. Clinical findings, sonographic features, differential diagnosis, further investigations, intrapartum management, postnatal complications, and histopathological characterization of this rare oral cystic mass are discussed and the recent literature is reviewed.

## 2 Case report

**Figure 1:**
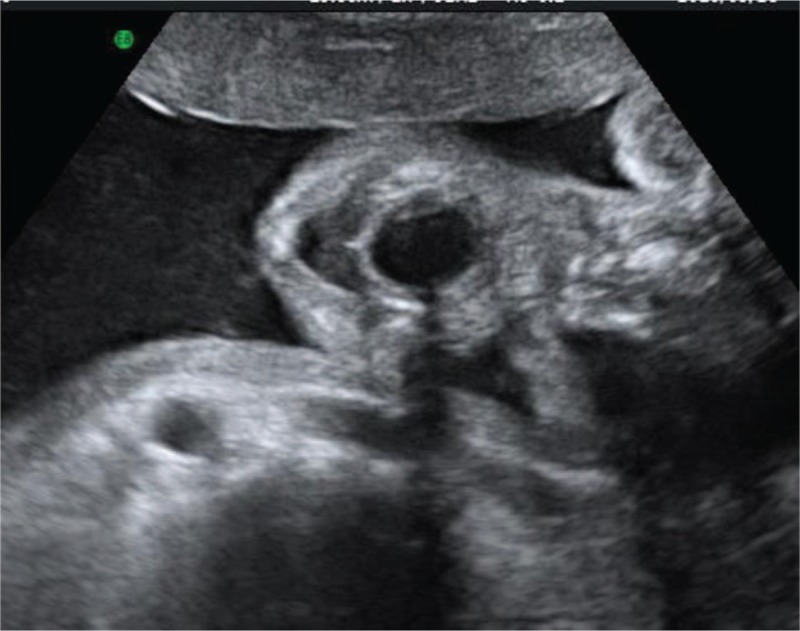
Axial view of the fetal mouth at 22 weeks of gestation shows an anechoic mass.

**Figure 2:**
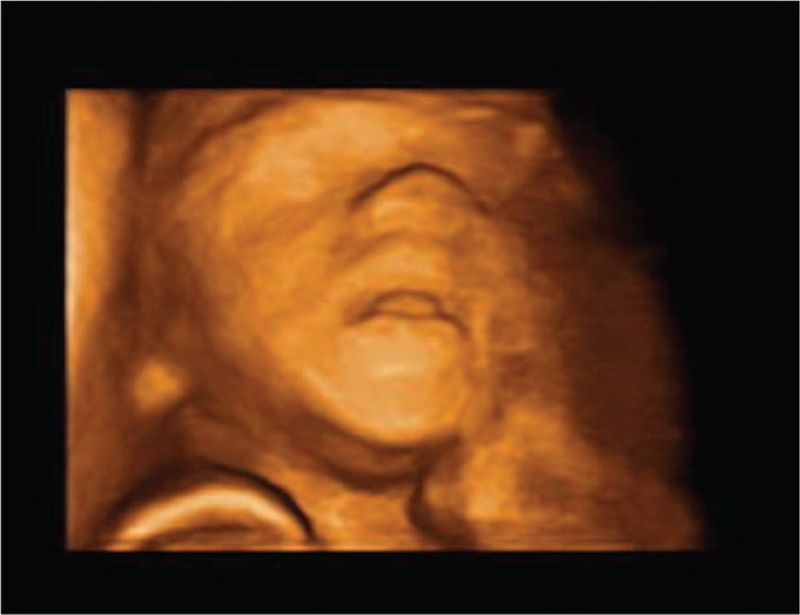
Three-dimensional image shows a slightly opened mouth and the mass is not protruding outside of the oral cavity.

**Figure 3:**
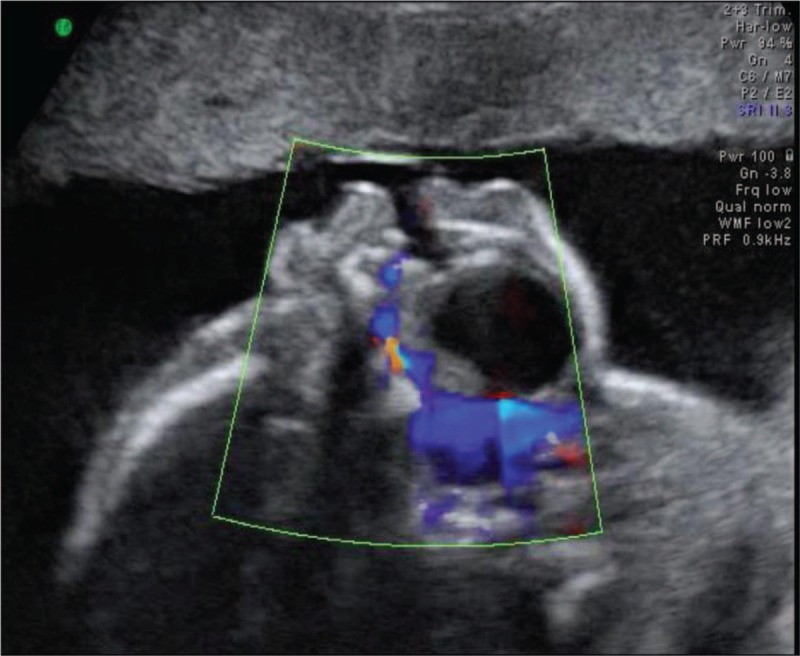
Color Doppler imaging shows the patency of the nasal/nasopharynx, hypopharynx, and trachea.

**Figure 4:**
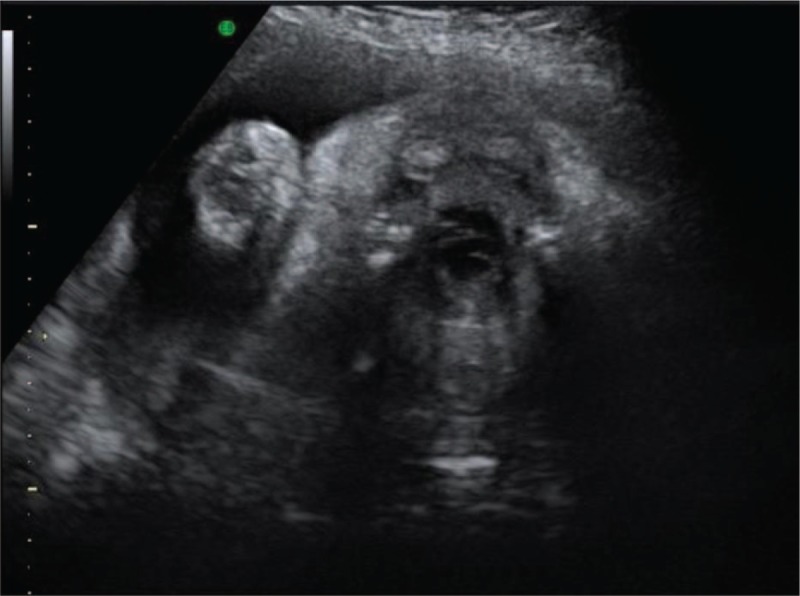
Follow-up ultrasound examination at 31 weeks of gestation. A tiny septum in the cyst can be seen.

A 36-year-old woman, G2P1, with an uneventful pregnancy, presented to Peking Union Medical College Hospital at 22 weeks’ gestation for routine second trimester screening. An ultrasound examination showed the presence of an oral cystic lesion, which measured 18 × 15 × 15 mm. The lesion was posterior to the mandible and attached to the inferior aspect of the tongue in a male fetus (Fig. 1). No blood flow signal was observed through the cyst. The lesion appeared to be moving in conjunction with the tongue and did not protrude outside of the oral cavity. The fetal mouth was slightly open for the duration of the ultrasound scan and was shown by 3-dimensional ultrasound in the surface render mode (Fig. 2). Color Doppler flow imaging (CDFI) showed fetal deglutition, with clear evidence of transoral amniotic fluid flow around the cyst and in the upper respiratory tract (Fig. 3). This indicated patency of the fetal airway. The fetal stomach was visualized and the amniotic fluid volume was in the normal range. This finding indicated normal swallowing function and the cystic mass did not obstruct the esophagus. A sonographic anatomical survey showed no other anomalies of the fetus. Follow-up ultrasound examinations at 24 and 31 weeks of gestation showed a progressive reduction in size of the lesion and absence of polyhydramnios. Tiny internal septation was observed (Fig. 4). However, the cyst eventually disappeared in follow-up examinations at 35 and 37 weeks of gestation.

A male newborn who weighed 3480 g was delivered vaginally, with Apgar scores of 10 and 10 at 1 and 5 minutes, respectively. The neonate had no feeding or breathing difficulty. No major oral cyst was identified in the tongue at a clinical and instrumental examination. We followed the child to 6 years after birth. The child had normal growth and development, with no recurrence of the cyst. The study was approved by the Institute Research Ethic Committee of Peking Union Medical College Hospital. The patient has provided informed consent for publication of the case.

## 3 Discussion

Congenital lingual cystic lesions are uncommon pathological entities,^[1]^ including a broad spectrum of disease, such as a ranula,^[2–5]^ foregut duplication cyst,^[6–8]^ cystic epignathus,^[9]^ thyoglossal duct cyst,^[10]^ and lymphangioma.^[11]^ Most of these lesions are benign. Ranulas have an incidence of 0.74%, and are usually secondary to a mucous leak following disruption of the sublingual salivary gland elements. Ranulas can also occur as a retention cyst derived from proximal expansion of a blocked duct.^[2–5]^ A foregut duplication cyst is a benign developmental anomaly that occurs along the foregut-derived alimentary tract with an incidence of 0.02%. This type of cyst most commonly arises in the thorax and abdomen.^[6,8]^ Foregut duplication cysts represent one third of gastrointestinal duplication cysts. Only 0.3% of these cysts arise in the tongue, typically from the anterior two thirds of the tongue.^[7]^ The cysts contain epithelial lining of ciliated respiratory, gastric, and squamous epithelium. They may originate in a defect of migration from the islands of endoderm of the primordial stomach in the 4-week embryo.^[12]^ Epignathus is a benign teratoma that is estimated to affect between 1 in 35,000 and 1 in 200,000 live births, and it accounts for 2% to 9% of all teratomas.^[9]^ Epignathus mostly involves the posterior nasopharynx, hard palate, or sphenoid bone. Occasionally, these lesions may have an intracerebral extension. These lesions mal also be associated with other malformations, including polyhydramnios, cleft palate, an abnormal mandibular structure, a bifid tongue or nose, branchial cleft cyst, and congenital heart disease.^[9]^ Epignathus may have cystic and solid components, and it commonly protrudes out of the fetal mouth. Thyroglossal duct cyst is a congenital developmental malformation that occurs because of incomplete obliteration of the thyroglossal duct with a general prevalence of 7%. This cyst type can be found along the path of the thyroglossal duct from the foramen cecum to the pyramidal lobe of the thyroid.^[10]^ Differential diagnosis among these entities might be difficult because most cystic lesions show a similar anechoic avascular ultrasound pattern. Three-dimensional techniques can help to provide potentially useful information on the size, location, extension, and composition of such masses.^[13]^ Definitive diagnosis can still only be made on the basis of histopathology of the lesion.

A search of PubMed showed only 18 cases of lingual cysts that were diagnosed prenatally. These cases included six ranulas, seven foregut duplication cysts, 2 thyroidglossal duct cysts, one lymphangioma, and one cystic epignathus. Prenatal recognition of such a tumor should prompt a thorough evaluation of the likelihood of airway tract and esophageal obstruction. CDFI and magnetic resonance imaging (MRI) provide the ability to visualize a fluid-filled oral cavity, oropharynx, nasopharynx, and trachea. Real-time ultrasound and MRI can demonstrate motion of the mass during swallowing. Normal amniotic fluid volume and visualization of stomach bubbles are helpful for determining if there is no distortion or compression of the esophagus. Routine follow-up should be scheduled bi-weekly to monitor growth of the lesion. Steelman et al^[14]^ reported a ranula that spontaneously resolved at 5 weeks of age. However, a cystic lesion that disappeared before birth has not been reported. In some of the reported cases, a considerable increase in lesion size required an intervention to relieve obstruction.^[4,9,15]^ Pires et al^[4]^ and George^[15]^ conducted ultrasound-guided aspiration of the cyst at 26 weeks of gestation; Deloison^[9]^ performed cyst puncture immediately prior to delivery. Prenatal decompression of the cyst can prevent postnatal airway obstruction, especially if carried out just before birth, which can also avoid the risk of hemorrhage from the cyst. Two of the reviewed cases underwent an extrauterine intrapartum treatment procedure with aspiration of the cyst to secure the airway of the infant.^[16,17]^ This technique allows maintenance of uteroplacental gas exchange in the newborn. The definitive treatment for these tumors is postnatal surgical resection.

## 4 Conclusion

We report a case of a relatively small cyst with spontaneous in utero resolution. A lingual cyst usually has a good prognosis. When a lingual cyst is prenatally diagnosed, the patency of the airway tract and esophagus should be evaluated. Prenatal aspiration of the cyst or the extrauterine intrapartum treatment procedure facilitates a successful outcome for large lingual cysts.

## Author contributions

**Investigation:** Hua Meng, Xining Wu.

**Methodology:** Hua Meng.

**Software:** Zihan Niu.

**Writing – original draft:** Yixiu Zhang, Hua Meng.

**Writing – review & editing:** Yixiu Zhang, Yunshu Ouyang, Hua Meng.
